# The clinical and cost effectiveness of steroid injection compared with night splints for carpal tunnel syndrome: the INSTINCTS randomised clinical trial study protocol

**DOI:** 10.1186/s12891-016-1264-8

**Published:** 2016-10-06

**Authors:** Linda S. Chesterton, Krysia S. Dziedzic, Danielle A. van der Windt, Graham Davenport, Helen L. Myers, Trishna Rathod, Milica Blagojevic-Bucknall, Sue M. Jowet, Claire Burton, Edward Roddy, Elaine M. Hay

**Affiliations:** Arthritis Research UK Primary Care Centre, Research Institute for Primary Care and Health Sciences, Keele University, Keele, Staffordshire ST5 5BG UK

**Keywords:** Carpal Tunnel Syndrome, Boston Carpal Tunnel Questionnaire, Night splints, Corticosteroid injection, Randomised clinical trial, Protocol

## Abstract

**Background:**

Patients diagnosed with idiopathic mild to moderate carpal tunnel syndrome (CTS) are usually managed in primary care and commonly treated with night splints and/or corticosteroid injection. The comparative effectiveness of these interventions has not been reliably established nor investigated in the medium and long term. The primary objective of this trial is to investigate whether corticosteroid injection is effective in reducing symptoms and improving hand function in mild to moderate CTS over 6 weeks when compared with night splints. Secondary objectives are to determine specified comparative clinical outcomes and cost effectiveness of corticosteroid injection over 6 and 24 months.

**Method/Design:**

A multicentre, randomised, parallel group, clinical pragmatic trial will recruit 240 adults aged ≥18 years with mild to moderate CTS from GP Practices and Primary-Secondary Care Musculoskeletal Interface Clinics. Diagnosis will be by standardised clinical assessment. Participants will be randomised on an equal basis to receive either one injection of 20 mg Depo-Medrone or a night splint to be worn for 6 weeks. The primary outcome is the overall score of the Boston Carpal Tunnel Questionnaire (BCTQ) at 6 weeks. Secondary outcomes are the BCTQ symptom severity and function status subscales, symptom intensity, interrupted sleep, adherence to splinting, perceived benefit and satisfaction with treatment, work absence and reduction in work performance, EQ-5D-5L, referral to surgery and health utilisation costs. Participants will be assessed at baseline and followed up at 6 weeks, 6, 12 and 24 months. The primary analysis will use an intention to treat (ITT) approach and multiple imputation for missing data. The sample size was calculated to detect a 15 % greater improvement in the BTCQ overall score in the injection group compared to night-splinting at approximately 90 % power, 5 % two-tailed significance and allows for 15 % loss to follow-up.

**Discussion:**

The trial makes an important contribution to the evidence base available to support effective conservative management of CTS in primary care. No previous trials have directly compared these treatments for CTS in primary care populations, reported on clinical effectiveness at more than 6 months nor compared cost effectiveness of the interventions.

**Trial registration:**

Trial registration: EudraCT 2013-001435-48 (registered 05/06/2013), ClinicalTrials.gov NCT02038452 (registered 16/1/2014), and Current Controlled Trials ISRCTN09392969 (retrospectively registered 01/05/2014).

**Electronic supplementary material:**

The online version of this article (doi:10.1186/s12891-016-1264-8) contains supplementary material, which is available to authorized users.

## Background

Carpal tunnel syndrome (CTS) is caused by compression of the median nerve as it passes through the carpal tunnel [1]. CTS leads to difficulties with day-to-day tasks and reduces capacity to work impacting on quality of life and general health [2–4]. Incidence rates in the general population have been estimated at 61–120 per 100,000 women and 35–60 per 100,000 men per year [5].

CTS is characterised by pain, paraesthesia and numbness in the median nerve distribution; usually worse at night causing sleep disruption, and may be alleviated by shaking the hand. Over half of patients have bilateral symptoms and the age distribution is bimodal peaking in the 50–54 and 75–84 age groups [1, 5–8].

There is no consensus on the best treatment for CTS, especially in primary care [9]. Surgical decompression is often considered the definitive solution to resolve symptoms in severe or unresponsive cases. Systematic review evidence comparing surgical with non-surgical treatments concluded that surgery produced superior results to splinting, but evidence for the benefit of surgery over corticosteroid injection was unclear [10]. In patients who have surgery up to 8 % are left with worse or unimproved symptoms [11].

The most commonly used treatments for mild/moderate CTS in primary care are splinting, anti-inflammatory medication and local corticosteroid injection [9, 12]. Systematic reviews of these treatments [9, 13–15] draw similar conclusions of moderate evidence in the short term for nocturnal hand bracing/splinting being more effective than no therapy (relative risk for overall improvement in symptom and function = 4.00; 95 % CI, 2.34–6.84) with limited evidence of medium term effects [9] and moderate to strong evidence for the effectiveness of corticosteroid injections over placebo (relative risk in terms of success rate: 2.58; 95 % CI, 1.72–3.87) in the short term [9]. In one primary study [16] of corticosteroid injections, higher doses (60 mg) appeared more effective than lower doses (20 or 40 mg) in the medium term (success rates for high dose 73 % versus 53-56 %) although the benefits were not maintained in the long term. Two injections of 15 mg steroid did not provide any additional clinical benefit over a single treatment [9]. Review authors conclude that many of the included trials had a high risk of bias and recommended that more robust trials are needed to compare treatments and ascertain the medium to long-term benefits [9, 13–15].

The effects of corticosteroid injection and splinting have been compared in two small trials, neither of which were carried out in a primary care setting. Sevim and colleagues showed superior effects of splinting over corticosteroid injection (*n* = 30) [17] and Ucan et al. found no significant differences in clinical outcome when comparing splinting (23 hands) to a combination of injection with splinting (23 hands) although both groups improved [18]. Robust inferences cannot be made from these studies.

We have therefore designed and are currently implementing a prospective randomised clinical trial to directly compare the clinical and cost-effectiveness of night splinting and corticosteroid injections as primary care management interventions for CTS.

## Methods

### Aim

The primary objective of the INSTINCTS trial is to investigate whether a corticosteroid injection is clinically effective in reducing symptoms and improving function in the short term (6 weeks) compared to a resting night splint in people consulting with mild to moderate carpal tunnel syndrome in primary care.

The secondary objectives are to examine clinical and cost effectiveness in the medium term (6 months) of a single corticosteroid injection compared to night splinting, from a healthcare (NHS) and personal social services perspective as well as from a societal perspective to include the differences in productivity loss. In the longer term (24 months) we will examine differences in symptoms, function, work absence, and other health care resource use, in particular, referral for CTS surgery.

### Design

The trial is a randomised, multicentre, open label, parallel group, pragmatic clinical trial comparing a single injection of Methylprednisolone Acetate (as 20 mg of Depo-Medrone 40 mg/ml) into the carpal tunnel versus 6 weeks of night splinting in patients with mild to moderate CTS.

### Eligibility criteria

The eligibility criteria (Table 1) are designed to select a relatively homogeneous group of patients with CTS, suitable for both splinting and local injection and who do not require immediate onward referral for surgery. For participants with bilateral CTS a study hand will be designated by the participant based on the most severe symptoms. Patients will not be allowed to enter the trial more than once.

**Table 1:** Eligibility criteria

Inclusion criteria	Exclusion criteria
Male or female aged ≥ 18 years	Corticosteroid injection or night splints for CTS in the affected wrist within preceding 6 months
A clinical diagnosis of unilateral or bilateral CTS as made by a GP or trained clinician according to the diagnostic criteria	Severe CTS exhibiting constant numbness or pain, constant sensory loss, severe Thenar muscle atrophy or symptom severity which requires the patient to be referred for a surgical opinion
Mild (e.g. intermittent paraesthesia) or moderate (e.g. constant paraesthesia, reversible numbness and/or pain) severity CTS of idiopathic nature	Any previous surgery on the affected wrist (or study wrist in the case of bilateral symptoms)
Symptom duration of episode of at least 6 weeks	Clinical suspicion of local or systemic sepsis or infection
Written informed consent provided by the patient, prior to any trial specific procedures	Current or previous infection of the affected wrist
	Trauma to the affected hand requiring surgery or immobilisation in the previous 12 months
	Unable to tolerate the study interventions
	Unable to understand and complete self-report questionnaires written in English
	Inter-current illness including, but not limited to: • poorly controlled thyroid disease • poorly controlled diabetes mellitus • vibration-induced neuropathy • inflammatory joint disease • suspected complex neurological conditions • any other severe medical illness which in the opinion of the local Principal Investigator (or other authorised clinical delegate) precludes trial participation
	Pregnant or lactating females
	Receiving anticoagulants
	Any history of hypersensitivity to Depo-Medrone or any of its excipients
	Allergy to any of the splint materials
	Known abuse of drugs or alcohol
	Involved in on-going litigation cases for their condition

### Inclusion criteria

The study population will consist of adults aged 18 years and over with a new episode of primary idiopathic mild to moderate CTS which has been present for more than 6 weeks. Clinical diagnosis will be made by a GP or trained clinician and standardised based on presenting symptoms, clinical history and physical tests using criteria developed as part of a consensus survey of GPs from the UK Primary Care Rheumatology Society [12]. Mild CTS is defined as intermittent paraesthesia in the distribution of the median nerve and moderate as constant paraesthesia, reversible numbness and/or pain of idiopathic nature [19].

### Exclusion criteria

Participants will be ineligible for the trial if they have received either a corticosteroid injection or night splints for CTS within preceding 6 months or had previous surgery in the affected wrist (or study wrist in the case of bilateral symptoms), severe CTS exhibiting constant numbness or pain, constant sensory loss, severe thenar muscle atrophy or symptom severity which requires the patient to be referred for a surgical opinion. Other exclusion criteria are listed in Table 1.

### Recruitment, screening process and enrolment

Participants will be recruited from up to 50 sites including; GP practices, primary-secondary care musculoskeletal interface clinics throughout the UK. Research sites will have received NHS permission and trial specific training covering the interventions and trial administrative procedures (Additional file 1).

### General practice recruitment

For patients consulting in general practice, who are diagnosed with mild-moderate CTS and are eligible to participate, information about the trial will initially be provided verbally by their GP. Interested eligible patients will be given a participant information leaflet. Patients will be asked to give written consent to provide their personal details to Keele University. This will permit contact by a Clinical Research Network Nurse who will provide a full verbal explanation of the trial for the patient to consider. This will include detailed information about the rationale, design and personal implications of the trial. The patients will be advised by the GP that consent to contact does not oblige them to take part in the trial. For patients willing to participate, a second appointment for randomisation and treatment with the treating clinician will be made (within a minimum of 24 h and a maximum of 4 weeks’ time). On attendance at their scheduled second appointment, the patient’s GP will check for continued eligibility for inclusion and gain written informed consent to study participation from those eligible patients willing to take part in the study.

### Primary/Secondary care musculoskeletal interface clinics recruitment

For patients consulting at musculoskeletal interface or similar clinics based in secondary care, patients will be informed verbally about the trial by a study clinician following an initial clinical assessment. Eligible patients who are interested will be provided with an information leaflet. When the patient has had adequate time to read the information, a study clinician will give full details of the trial and answer any questions. Those patients willing to take part will be required to provide full written consent. A list of sites at which patients will be recruited is provided as Additional file 1.

### Randomisation

Prior to randomisation all participants will complete the baseline questionnaire, which includes questions regarding sociodemographic characteristics, outcome measures and potential prognostic factors. Participants will be randomised by the local clinician, in a 1:1 ratio via a remote web based randomisation system, which ensures the allocation sequence is concealed. The randomisation sequence is based on computer generated, random permuted blocks of sizes 2 and 4 and will be blocked by research site. Following completion of randomisation patients will be given a patient card detailing their treatment allocation, which they are asked to carry at all times during the first 6 weeks and to present it to medical staff should they be admitted to hospital during this time on the study.

### Blinding

Blinding of participants and practitioners is not possible due to the study design. Group allocation will however be concealed to both parties prior to randomisation. Both primary and secondary outcomes are based on self-report questionnaires therefore there will be no investigator bias introduced at assessment stage. Analysis at the primary end point will be conducted blind to group allocation.

### Interventions

To reflect the consensus regarding usual practice for administering corticosteroid injection for CTS in primary care, participants randomised to injection will receive one injection of 20 mg of Depo-Medrone (from 40 mg/ml) via a disposable needle (23 or 25G) and syringe. This will be inserted at the wrist to infiltrate the carpal tunnel. A sterile ‘no-touch’ technique will be used and the addition of local anaesthetic is not permitted. The patient will sit with the hand placed palm upwards in a neutral or slightly extended wrist position. The skin is cleaned according to local policy and the needle inserted between the proximal and distal skin creases at the wrist, preferably on the ulnar side of the Palmaris longus which lies over the median nerve. The needle is angled at 45 degrees distally to enter the carpal tunnel. Occasionally, due to anatomical variations, e.g. large superficial veins, the injection may be given on the radial side of the Palmaris longus. Injections into the palm of the hand are not permitted as there are rare reports of ischaemic necrosis of the fingertips presumably due to the needle penetrating the palmar arterial arch. Participants will be advised to wait for 30 min following injection and to rest the injected arm for 48 h. They will be given two Arthritis Research UK patient leaflets: “Carpal tunnel syndrome” and “Local corticosteroid injections”. No other additional types of therapy are advised during the 6 week treatment period except for simple analgesia prescribed by the treating clinician or bought over the counter (e.g. paracetamol, NSAIDS), information about which will be captured via the 6 week questionnaire.

Participants randomised to night splints will receive a splint to wear at night for 6 weeks. The splint immobilizes the wrist in a neutral or slightly extended position (20 degrees from neutral) in order to avoid movement of the wrist, which increases carpal tunnel pressure [20]. The Promedics® Beta Wrist Brace (with CE Marking) is designed to avoid restriction at metacarpophalangeal joints and thumb, and the binding will ensure comfort to enhance adherence to splint wearing. Each splint will be fitted according to the size of the participant’s hand and arm using standard splints of differing sizes. The wrist angle will be in neutral position (between 0 and 20 degrees) to reduce carpal tunnel pressures. No other types of therapy will be permitted during the 6 weeks, except simple analgesia prescribed by the treating clinician or bought over the counter (e.g. paracetamol, NSAIDS), which will be captured in the 6 week questionnaire.

Participants will be shown how to fit and remove the wrist splint according to a standardised trial protocol. Adherence will be encouraged and reinforced by verbal instruction from the clinician on how and when to use the splint and this will be supported by written information, detailing care, fitting and use of the splint. Participants will be instructed to perform gentle range-of-motion exercises when removing the splint to prevent stiffness. They will also be given two Arthritis Research UK patient leaflets: “Carpal tunnel syndrome” and “Splints for arthritis of the hand and wrist”.

For participants with bilateral CTS, the non-study hand will be treated according to normal clinical protocols in use at the research site.

### Outcome measures and endpoints

The primary endpoint will be at 6 weeks post randomisation and secondary endpoints will be at 6, 12 and 24 months post randomisation. Table 2 explains which outcomes are collected at the different time-points. The primary outcome measure will be the overall score of symptom severity and limitations in hand function as assessed by the BCTQ [21] which has previously been shown to be highly reproducible, internally consistent, valid, and responsive to clinical change in CTS patients [22, 23]. The BCTQ is a disease specific questionnaire referring to a typical 24 h period in the last 2 weeks. It consists of two subscales: symptom severity scale (SSS: 11 items) and function status scale (FSS: 8 items), both scored on 1–5 point scales, with final scores for each dimension calculated as a mean score between 1 and 5. The overall score is calculated as the mean of all 19 items. Higher scores correlate with more severe symptoms and functional impairment.

Secondary outcome measures include; BCTQ symptom severity and function status subscales [21], hand-wrist symptom intensity (0–10 numerical rating scale), interrupted sleep, [24] self-reported adherence to splinting where indicated (Likert scale modified from previous research [25]), patients’ perceived benefit and satisfaction with treatment (Likert scale modified from previous research) [25], impact of CTS on work and other activities (including work absence and reduction in work performance measured by a 0–10 rating scale), referral for surgery, surgery, general health (EuroQoL EQ-5D-5L) [26], quality adjusted life years, health care utilisation and patient incurred costs, and use of co-interventions such as supplements and analgesia. Participants’ expectations regarding treatment response [25] and presence of bilateral CTS symptoms will be explored as potential effect modifiers.

**Table 2:** Outcome measures and data collection time points

Baseline measures	Description	Baseline	6 week follow-up	6 month follow-up	12 month follow-up	24 month follow-up
Demographics	gender, date of birth	✓	✓	✓	✓	✓
Previous CTS episodes	number of episodes and treatments	✓				
Current CTS episode	location and duration	✓				
Participant preference and expectations for treatment	Likert Scale adapted from previous research	✓				
General Health	Self-reported health and comorbidities	✓				
Primary outcome measure						
Hand/wrist pain and function	Boston CTS questionnaire	✓	✓	✓	✓	✓
Secondary outcome measures						
Hand/wrist pain intensity	NRS (0–10) over last 24 h	✓	✓	✓	✓	✓
Interrupted Sleep	Estimation of interruption to sleep scale	✓	✓	✓		
Adherence with splinting	Scales adapted from previous research		✓			
Other Treatments received	Self-reported referral to surgery and prescribed analgesia		✓	✓	✓	✓
Satisfaction and experience	Likert scales adapted from previous research		✓			
Health economic outcomes						
Health related quality of life	EuroQoL:EQ-5D-5L	✓	✓	✓	✓	✓
Employment	Current employment status and employer support	✓	✓	✓	✓	✓
Performance at work	How performance at work is affected NRS 0-10	✓	✓	✓	✓	✓
Work absence	Self-report days absent from work	✓	✓	✓	✓	✓
Healthcare utilisation	Primary care consultations			✓	✓	✓
	Secondary care contacts including investigations					
	Prescribed medications					
	Use of private healthcare					
Patient-incurred costs and use of co-interventions	Over-the-counter medications or interventions			✓	✓	✓

Schedule of events

### Adverse events

Although Depo-Medrone is not specifically licensed for use in CTS, it has been widely used for many years in standard practice in both primary and secondary care and has a very well established and understood safety profile [27–29]. It is being used within the injection protocol in accordance with the guidance given in the BNF [28] for local inflammation in soft tissues and expert opinion provided in Map of Medicine [27] regarding the use of corticosteroid for CTS. The incidence of adverse predictable undesirable side-effects associated with the use of corticosteroids correlates with the relative potency of the drug, dosage, and timing of administration and duration of treatment, and therefore based on the dosage to be used in this study, there is no requirement within this study to record non-serious adverse events beyond normal clinical practice. Where a Serious Adverse Event (SAE) or Suspected Unexpected Serious Adverse Reaction (SUSAR) occurs, reporting procedures are in place that are in accordance with good clinical practice guidance and the requirements specified by the MHRA. All SAEs will be considered by the external monitoring committees.

### Data collection methods

All participants enrolled in the study will be asked to complete a paper questionnaire at the baseline clinical appointment and again at 6 weeks, 6, 12 and 24 months when a questionnaire will be posted to them. Questionnaires will capture data on all outcome measures. If the questionnaire is not returned within 2 weeks of initial mailing, a reminder will be sent (text message, e-mail, or telephone call). If the questionnaire is still not returned, a second follow-up questionnaire will be sent 4 weeks later. For non-responders, minimum data collection (MDC) to capture the primary outcome measures will be undertaken by a research nurse by telephone at 2 weeks after mailing of the second questionnaire. Where a participant is not able to be contacted by telephone within three working days, a postal MDC questionnaire will be sent. Patients who do not respond or are not contacted by telephone will still be sent subsequent questionnaires at further follow-up time points unless they request not to.

**Fig. 1:**
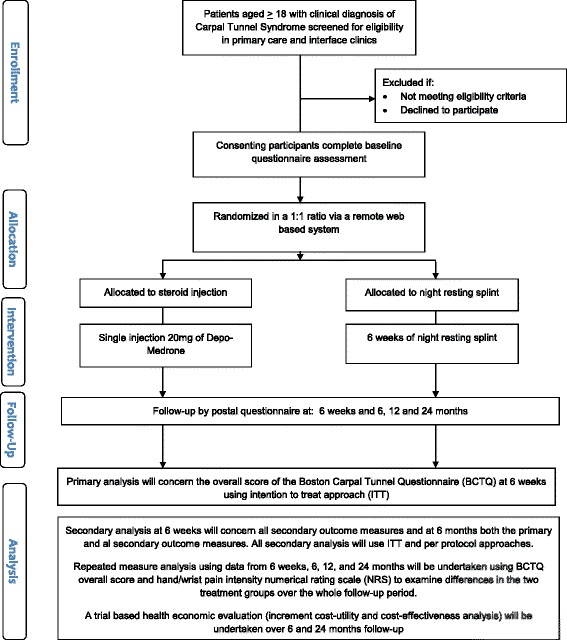
Flow of participants through the trial

The flow events as participants proceed through the trial is outlined in (Fig. 1) and the the timing of key events outlined in Table 3.

**Table 3:** Participant timeline

Visits	Screening	Baseline and intervention Day 0	6 week follow-up	6 month follow-up	12 month follow-up	24 month follow-up
Diagnosis	✓					
Eligibility Screening	✓					
*GP pathway only:* Written Informed Consent for contact by Keele RN and completion of Consent to Contact Fax Form	✓					
*GP pathway only:* Keele RN telephone call to potential participant	✓					
*GP pathway only:* Confirmation of Eligibility and Randomisation		✓				
Informed consent by authorised local Investigator		✓				
Participant Baseline Data self-report Questionnaires		✓				
Web randomisation		✓				
Administration of study intervention		✓				
Participant self-report follow up Questionnaires			✓	✓	✓	✓

### Statistical analysis

#### Primary analysis at 6 weeks

Baseline characteristics will be summarized for the two treatment groups using appropriate descriptive statistics. The primary analysis will be conducted blind to treatment allocation and will be analysed on intention to treat (ITT) approach with all randomised participants retaining their original randomised group. Multiple imputation using chain equations [30] will be used to impute missing data. Sensitivity analyses will be performed to compare ITT results to those based on complete-case analysis.

Multiple linear regression will be used to obtain the mean estimates and 95 % confidence intervals for the difference in the BCTQ overall score at 6 weeks between the treatment groups adjusting for age at recruitment, sex, duration of symptoms, and index score at baseline.

#### Secondary analyses (outcomes up to 6 months)

Using similar methods described for the primary analysis, the effect of treatment will be estimated over the medium term (6 months post-randomisation) as measured by the BCTQ overall score, and also for the secondary outcome measures listed above recorded at 6 weeks and 6 months.

Per protocol analyses, based on self-report adherence to night splinting and use of a single injection, will be carried out. No interim analyses are planned.

Potential effect modifiers, i.e. participants’ expectations regarding treatment response and presence of bilateral CTS, will be explored through adding moderator*treatment interactions to the models estimating the primary outcome of symptom severity and function (acknowledging that interpretation of *p*-values will be placed in context of anticipated low power as well as the exploratory nature of the tests), and describing effects of treatment for subgroups of patients defined based on modifying variables.

### Sample size and calculation

Although previous studies using the BCTQ have provided information on the minimal important within-group change (0.23 points) there is limited evidence to underpin the definition of a minimum clinical important difference between the expected effects of injections versus night splinting for the primary outcome measure. Previously published RCTs have either examined the effectiveness of injection or night-splinting in patients with CTS, but very few have compared the two treatments. As both the treatments in this trial are active, we would expect a smaller difference between the treatment groups than if an active were compared to a control group, where a minimum of a 20 % difference might be expected. Hence, in order to detect a 15 % greater improvement, as measured by the BCTQ from an expected baseline value of approximately 2.9 points (scale 1–5, SD 1.0) [18, 31–33] in the injection group compared to night-splinting (i.e. a 0.9-point (30 %) reduction in the injection group versus a 0.45-point (15 %) reduction in the night splinting group, with pooled SD of 1.0, SMD 0.45), we would need complete data for 200 patients (100 in each treatment group) given approximately 90 % power and 5 % two-tailed significance. By adopting an initial sample size that would take account of 15 % loss of data (which we achieved in previous similar studies), we would need to recruit 120 participants per treatment group, 240 patients altogether. Through routine, 6-monthly examination of the follow-up rates, as part of the reporting for the Trial Steering and Data Monitoring Committees, we will be able to examine the deviation of the observed attrition rate from the expected 15 % factored into the sample size calculation.

### Outcomes across the 24-month follow-up period

A repeated measure analysis using data from the four follow-up points (6-week, 6-, 12-, and 24-months) will be undertaken with two key clinical outcomes (BCTQ overall score and hand/wrist pain intensity NRS) to examine differences in the two treatment groups over the whole follow-up period. Additionally, the cumulative number of participants who report on the self-complete questionnaires that they have been i) referred for CTS surgery or ii) undergone CTS surgery will be examined by treatment group.

All analyses will be performed using STATA 14. Statistical estimates will be accompanied by associated 95 % confidence intervals and all *p* values <0.05 considered to indicate statistical significance.

### Health economics

The base-case economic analysis will adopt an NHS and personal social services (PSS) perspective, as recommended by the NICE reference case for economic evaluations [34]. However, a sensitivity analysis will also be undertaken to consider a wider societal perspective, where all relevant costs are measured, including direct health care costs incurred within both public and private sectors, and the indirect costs outside the health care sector associated with productivity loss (absenteeism). The primary economics analysis will be a cost-utility analysis to enable comparisons to be drawn with other areas of health care; utility will be measured using quality-adjusted-life-year (QALY) values which will be derived from the EuroQoL EQ-5D-5L. A cost-effectiveness analysis will also be carried out using change in the total BCTS score as the measure of outcome. Results will be presented for the medium-term using data for the first 6 months and long-term, using data for all 24 months of follow-up.

Data on health care resource use, time off work and presenteeism/performance will be obtained from the participant questionnaires at 6, 12 and 24 months. Participants will be asked about primary and secondary care contacts, investigations, treatments and surgery over-the-counter purchases, and prescriptions. Health care resources associated with delivery of the interventions will also be determined. NHS care will be costed as national averages with inpatient and outpatient episodes costed using NHS reference costs [35, 36]. Due to the paucity of high-quality unit cost data for private health care consultations these data will be costed as the NHS equivalent. The British National Formulary (BNF) will be used to cost prescribed medication [28]. Costs of absenteeism from paid work will be estimated by multiplying the reported number of days off work by the average daily wage, stratified by hourly mean income according to gender, full/part-time work status and standard occupational classification (SOC 2010) [37]. The human capital approach to calculating productivity costs will be used, with the friction cost approach used in a sensitivity analysis.

Cost data alongside trials are invariably skewed. We will calculate 95 % confidence intervals (CIs) around differences in mean costs, EQ-5D scores, QALYs, and changes on the Boston CTS questionnaire using conventional parametric methods and non-parametric bootstrapping (5000 replications) [38]. The total QALYs per patient will be calculated using the area under the curve method, with adjustment for difference in baseline utility scores if required [39]. Multiple imputation will be employed where there is missing cost and/or outcome data.

The aim of the economic evaluation will be to estimate and compare the difference in costs of corticosteroid injection compared with night splinting and relate this to the difference in outcomes of injection versus splinting. An incremental approach will be used in the analysis, with differences in costs and QALYs expressed as an incremental cost-effectiveness ratio (ICER) - cost per additional QALY gained. Similarly for the cost-effectiveness analysis, we will analyse the incremental cost per 1-point improvement in total BCTS score. The robustness of the results will also be explored using deterministic sensitivity analysis. This will explore uncertainties in the trial based data itself, the methods employed to analyse the data and the generalisability of the results to other settings. Uncertainty in the confidence to be placed on the results of the economic analysis will be explored using probabilistic sensitivity analysis. Bootstrap samples are used to compose cost effectiveness/utility planes (which graphically show the variability in the data) and cost effectiveness acceptability curves are plotted to quantify, from the bootstrap data, the probabilities of the interventions being cost effective across a range of ceiling values (otherwise referred to as the willingness-to-pay threshold values).

### Timing of analysis

The primary analysis to examine the clinical and health economic outcomes will be blinded to treatment and completed after the final 6 month follow-up questionnaire has been received. The treatment allocation will be unblinded once this analysis has been completed in accordance with the agreed analysis plan. The analysis of the 12 and 24 month data will be analysed after the final 24 month follow-up questionnaire has been received and will not be blind to treatment allocation. No interim analyses will be performed.

## Discussion

This paper describes the design of pragmatic randomised trial which investigates the comparative clinical and cost effectiveness of corticosteroid injections and night splints in reducing symptoms and improving hand function in mild to moderate CTS over the short (6 weeks), medium (6 months) and long term (24 months). A number of issues have been addressed in the design of this study.

In terms of diagnosis there is a fundamental problem in a lack of an accepted “gold standard.” Furthermore CTS is routinely classified as mild, moderate or severe although criteria are not well established [19]. Many studies have been undertaken to evaluate diagnostic tools and tests for CTS, [40–45] most of which have been carried out in secondary care on patients with severe symptoms, using either neurophysiological testing, expert clinical opinion or surgical outcomes as reference standard. The American Academy of Orthopaedic Surgeons (AAOS) have published guidelines on the diagnosis and management of CTS and suggest that the most reliable approach for clinical diagnosis includes neurophysiological tests, structured history taking and physical symptoms coupled with the patient self-report hand diagram [29]. This is not universally accepted and there is considerable controversy as to the need for neurophysiological testing in carpal tunnel syndrome diagnosis. Neurophysiological studies are often used in secondary care especially prior to surgical intervention or in severe clinical presentations. However neurophysiological studies do not always correlate well with clinical severity and have not been shown to accurately predict outcomes for patients with mild to moderate CTS. Primary care access to nerve conduction studies is variable throughout the UK. As such these investigations are usually reserved for equivocal diagnoses and are not currently required routinely in this setting for mild/moderate cases to guide decisions regarding initial conservative treatments [46].

For the purposes of this trial it was therefore necessary to develop a tool to standardise the diagnosis of CTS presenting in Primary care. A Delphi technique study was undertaken to seek the opinions and usual practice of GP’s with an interest in musculoskeletal medicine. Forty five GPs with an interest in hand research were identified from the Primary Care Rheumatology Society. The opinions of panel members were sought through three rounds of questionnaires, distributed anonymously, using an online survey website between October 2011 and January 2012. A clinical consensus meeting was held at the end of the study to finalise the tool which was then disseminated for approval and any further comments. The consensus exercise resulted in a clinical diagnostic tool that can be used to standardise the diagnosis of CTS for both trial purposes and in clinical practice, designed specifically for use in primary care [12].

Eligibility criteria were defined to recruit a representative primary care population of mild to moderate CTS patients, protect patient safety and also to ensure maximum generalisability of the results to primary care. Accordingly we chose to include patients with well controlled diabetes and thyroid disease because. Routinely in general practice patients with well controlled diabetes and thyroid disease will be offered the study treatments.

Whilst conducting the trial in a setting that is very close to primary care is crucial to our research question and to optimizing the generalisability of the findings, we recognised the need to maximise recruitment and achieve realistic recruitment targets. Therefore we decided to use as recruitment sites, musculoskeletal services which receive direct referrals from multiple general practitioners who themselves do not inject CTS patients. By strictly applying the eligibility criteria we can demonstrate the study population is still representative of the target population.

Although corticosteroid injection is a commonly used primary care intervention, there may be potential risk of harm which includes median nerve damage, infection and tendon rupture. While the incidence is extremely low and evidence mostly based on case descriptions this risk in this trial will be limited by; restricting the intervention to a less powerful corticosteroid (20 mg of Depo-Medrone), limiting the number of permissible injections to one in the 6-week treatment period, excluding patients with injections in the past 6 months and careful clinical assessment to ensure exclusion of patients at risk of an adverse event. Adverse events associated with interventions will be monitored via patient report to their GPs, case report forms and patient self-report questionnaires. GPs will be trained in reporting of serious adverse events and SUSARs as part of training in the study protocol.

### Clinical relevance of this trial and importance of the question

This paper describes the rationale and design for the first randomised pragmatic trial that aims to determine the comparative effectiveness of two commonly used interventions for patients with mild to moderate CTS. No previous trials have directly compared these treatments for CTS in primary care populations or reported on clinical effectiveness beyond 6 months. Comparative cost effectiveness of the interventions has not been investigated. The proposed trial will make an important contribution to the evidence base available to support effective conservative management of CTS in primary care and will inform both patient management and future research for treatment options for CTS.

## Additional file

Additional file 1: INSTINCTS list of recruitment sites. (DOCX 17 kb)

## Abbreviations

AAOS: American Society of Orthopaedic Surgeons
BCTQ: Boston Carpal Tunnel Questionnaire
BNF: British National Formulary
BSSH: British Society of Surgery for the Hand
CTS: Carpal tunnel syndrome
CTU: Clinical trials unit
EQ5d-5L: EuroQol Health related quality of life questionnaire
FSS: Functional symptoms subscale
GCP: Good clinical practice
GP: General practitioner
IMP: Investigational medicinal product
ITT: Intention to treat
MDC: Minimum data collection
MHRA: Medicines and Healthcare Products Regulatory Agency
NHS: National Health Service (UK)
NICE: National institute for health and clinical excellence
NRS: Numerical rating scale
NSAIDs: Non-steroidal anti-inflammatory drugs
PCRS: Primary Care Rheumatology Society
PPI: Patient and Public Involvement
QoL: Quality of life
QUALY: Quality-adjusted life-year
RCT: Randomised clinical trial
RN: Research nurse
SAE: Serious adverse event
SAR: Serious adverse reaction
SD: Standard deviation
SPC: Summary of product characteristics
SSS: Pain severity symptom subscale
SUSAR: Suspected unexpected serious adverse reaction
TSC: Trial Steering Committee
WHO: World Health Organization
