# Supplementary material for: The clinical and cost effectiveness of steroid injection compared with night splints for carpal tunnel syndrome: the INSTINCTS randomised clinical trial study protocol
Source: BMC Musculoskelet Disord. 2016 Oct 6;17:415. doi: 10.1186/s12891-016-1264-8 (PMC5053124; doi:10.1186/s12891-016-1264-8)
Supplement: Additional file 1: — INSTINCTS list of recruitment sites. (DOCX 17 kb) [file 12891_2016_1264_MOESM1_ESM.docx]

| **INSTINCTS recruitment sites** |
| --- |
| The Haywood Hospital, Stoke-On-Trent |
| Pennie Musculoskeletal Partnership, Oldham |
| Market Harborough Medical Centre, Market Harborough |
| Eccleshill Community Hospital, Bradford |
| Greyfriars Therapy Centre, Stafford |
| Wrekin Community Clinic, Telford |
| Sandwell and West Birmingham Hospitals NHS Trust, Birmingham City Hospital |
| Wolstanton Medical Centre, Newcastle-under-Lyme |
| Higherland Surgery, Newcastle-under-Lyme |
| Queens Square Medical Practice, Lancaster |
| Newport Pagnell Medical Centre, Newport Pagnell |
| Thurmuston Health Centre, Leicester |
| The Bondgate Surgery, Northumberland |
| Grovsenor Medical Centre, Tunbridge Wells |
| Waterloo Medical Centre, Lancashire |
| Rother House Medical Centre, Stratford-upon-Avon |
| York Medical Group, York |
| Kiltearn Medical Centre, Nantwich, Cheshire |
| Ashfields Primary Care Centre, Sandbach, Cheshire |
| Claughton Medical Centre, Merseyside |
| The Health Centre @ Glyndwr University Wrexham |
| Vauxhall Primary Health Centre, Liverpool |
| Pennine-Lancashire Integrated Musculoskeletal Service , Accrington PALS, Accrington |
| Priory Medical Centre, York |
| Great Sutton Medical Centre, Ellesmere Port, Cheshire |
| Aireborough Family Practice, Leeds |
